# From pathogenesis to response: occurrence mechanisms, host resistance mechanisms, and management strategies of apple ring rot

**DOI:** 10.3389/fmicb.2026.1897243

**Published:** 2026-09-03

**Authors:** Yue Chen, Zhu Li, Rongjing Cai

**Affiliations:** 1 Key Laboratory for Research on Zhaotong Apple Resources, College of Agronomy and Life Sciences, Zhaotong University, Zhaotong, China; 2 Yunnan Key Laboratory of Smart Villages and Agri-Cultural-Tourism Integration, Zhaotong University, Zhaotong, China

**Keywords:** apple ring rot, *Botryosphaeria dothidea* (*B. dothidea*), control measures, pathogenesis, resistance mechanism

## Abstract

Apple ring rot, caused by *Botryosphaeria dothidea* (*B. dothidea*) and *Botryosphaeria kuwatsukai* (*B. kuwatsukai*), is an economically important fungal disease of apple that primarily damages apple branches and fruits. This article comprehensively reviews the research progress on apple ring rot, covering its disease symptoms (on branches, fruits and leaves), pathogenesis (etiology and occurrence regularity), host resistance mechanisms (basal resistance and induced resistance), and integrated control strategies (agricultural, physical, chemical and biological control). Meanwhile, it analyzes the key problems existing in current research and prospects future research directions. This review aims to provide a systematic theoretical basis and technical implications for the effective prevention and control of apple ring rot, and ultimately promote the sustainable and healthy development of the apple industry.

## 1 Introduction

Apple (*Malus domestica*) is a globally important fruit crop with extensive cultivation and high economic value. Its fruits are rich in vitamins, minerals, antioxidants and other nutrients, occupying an important position in the global agricultural economy and human dietary structure (Pickson et al., 2026; Girotto et al., 2025; Kumari et al., 2024; Zhao et al., 2026). However, apple production is threatened by various diseases, among which apple ring rot, also known as apple rough bark disease or apple white rot, was first reported in Japan in the early 20th century and subsequently discovered in South Korea, China, the United States and other countries. It has a major biotic constraint on apple production in major planting regions worldwide (Brown and Britton, 1986; Ogata et al., 2000; Tang et al., 2012; Vasic et al., 2013; Ilyukhin et al., 2022).

Due to the latent infection state formed by pathogen–host interactions, the pathogen can invade fruits at the young fruit stage and remain latent for several months until symptoms appear during fruit ripening or storage. This characteristic makes early diagnosis and control of the disease extremely difficult (Kohn and Hendrix, 1983; Marsberg et al., 2017; Parker, 1993). Consequently, the “invisible” nature of the infection during the growing season creates a critical window where the pathogen establishes itself without triggering host defenses, rendering traditional scouting and reactive control measures largely ineffective. For a long time, academic research on apple ring rot has undergone a development process from traditional disease description and pathogen identification to modern molecular mechanism analysis and green control technology development (Kang et al., 2009; Li M. et al., 2024; Tong et al., 2026). Moreover, research on apple ring rot has long focused on the occurrence regularity and chemical control of the disease (Liu et al., 2022; Dong X. L. et al., 2021; Du et al., 2024). Although traditional chemical control can effectively control the occurrence of the disease in the short term, long-term and extensive use of chemical fungicides not only leads to the rapid evolution of pathogen resistance, but also causes a series of problems such as pesticide residues, environmental pollution and food safety, which seriously contradict integrated green pest management (IPM) and sustainable apple orchard production systems (Na et al., 2009; Zhong-Gang et al., 2005; Wang L. et al., 2021). This reliance on chemical intervention represents a “pesticide treadmill” where short-term efficacy is increasingly traded for long-term sustainability and regulatory compliance.

With the development of molecular marker technology and multi-omics, the taxonomic status of the pathogen has been gradually clarified, and the pathogenic factors of the pathogen and the resistance mechanism of the *M. domestica* have been increasingly investigated (Wang et al., 2018; Wang et al., 2024; Xin et al., 2023). In recent years, the emergence of advanced technologies such as cross-kingdom RNA interference (RNAi), host-induced gene silencing (HIGS), new natural fungicides and precise disease resistance molecular markers has provided new ideas for the control of apple ring rot (Du et al., 2024; Li P. et al., 2024; Weiberg et al., 2013; Yu et al., 2024). However, despite significant progress in related research, the control of apple ring rot still faces many challenges, such as the difficulty of early diagnosis caused by the latent infection characteristic of the pathogen, the problem of pathogen resistance caused by long-term use of chemical fungicides, and the insufficient field stability of biological control technologies, most of whose efficacy data are merely obtained from laboratory and detached-fruit assays rather than large-scale orchard-based trials (Huang et al., 2021; Fan et al., 2019; Sun et al., 2023). Current management strategies often operate in silos, failing to integrate these disparate technological advances into a cohesive framework that addresses the pathogen’s lifecycle from latent colonization to symptom outbreak.

Given the persistent challenges hindering sustainable management of apple ring rot, it is of critical importance to systematically collate state-of-the-art research advances and synthesize unresolved research gaps in this field. Most prior reviews analyze pathogen infection or resistance mechanisms in isolation rather than establishing a complete holistic research framework. Accordingly, the present review comprehensively summarizes existing findings covering disease symptomatology, pathogenesis, host resistance mechanisms, and integrated management strategies, while also outlining key unresolved questions and promising avenues for subsequent exploration.

## 2 Symptoms of apple ring rot

The symptoms of apple ring rot mainly appear on branches and fruits, and can also damage leaves, although leaf symptoms are relatively rare. Symptoms in different parts have obvious differences, and the disease development process presents typical stage characteristics (Liu et al., 2022; Xiang et al., 2024; Xue et al., 2021). However, a significant methodological limitation persists in the current literature. Most existing literature independently records lesion phenotypes on branches, fruits or leaves without unified comparative observation under consistent environmental gradients, restricting comprehensive understanding of the full disease symptom system. This fragmentation makes it difficult to correlate symptom severity across different host tissues, hindering the development of holistic disease forecasting models. Below we systematically describe the two core tissue-specific disease phenotypes of branches and fruits.

### 2.1 Symptoms on apple branches

**Figure 1:**
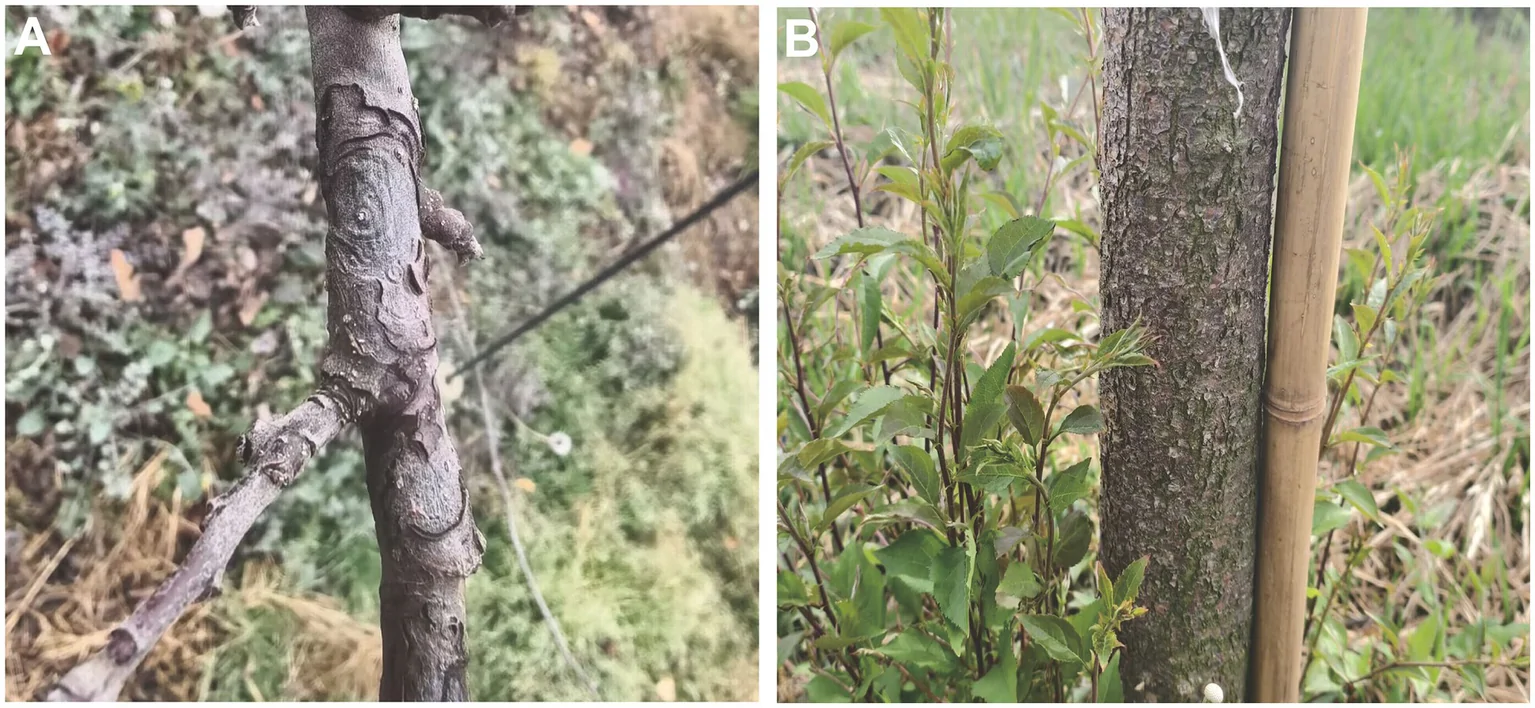
Symptoms of apple ring rot on branches. **(A)** Numerous nodular protrusions on the branch surface. **(B)** Rough and uneven branch epidermis showing the typical “rough bark” symptom.

Apple branches are the main overwintering site of the ring rot pathogen and the primary inoculum source of the disease. After infecting branches, symptoms typically develop centered on lenticels. At the initial stage of infection, small reddish-brown, water-soaked spots appear around the branch lenticels, and then the spots gradually expand to form reddish-brown nearly circular lesions with a measuring approximately 3–20 mm in diameter (Dong X. L. et al., 2021; Tang et al., 2012). These lesions are firm, with the central area gradually becoming raised and forming an obvious ring groove at the edge between diseased and healthy tissues, presenting a verrucous appearance (Dong B. Z. et al., 2021; Han et al., 2016) (Figure 1A). As the disease progresses, the lesions expand year by year, and lesions multiple lesions merge with each other may coalesce, resulting in rough and uneven branch epidermis, showing the typical “rough bark” symptom (Figure 1B). As the lesions continue to expand, severe infections may girdle branches, and impair vascular function, leading to reduced, tree vigor, leaf yellowing, branch dieback, and, in severe cases, plant death (Dong B. Z. et al., 2021; Miller, 2003). Beyond damaging host vascular tissue, mature lesions also develop *B. dothidea* conidiomata that serve as the primary inoculum source for subsequent infections (Liu et al., 2022). While these morphological changes are well-documented, there is a lack of quantitative data linking the extent of vascular occlusion to the specific physiological decline in tree vigor. Understanding this threshold is critical for determining the economic injury level and the optimal timing for pruning interventions.

### 2.2 Symptoms on apple fruits

**Figure 2:**
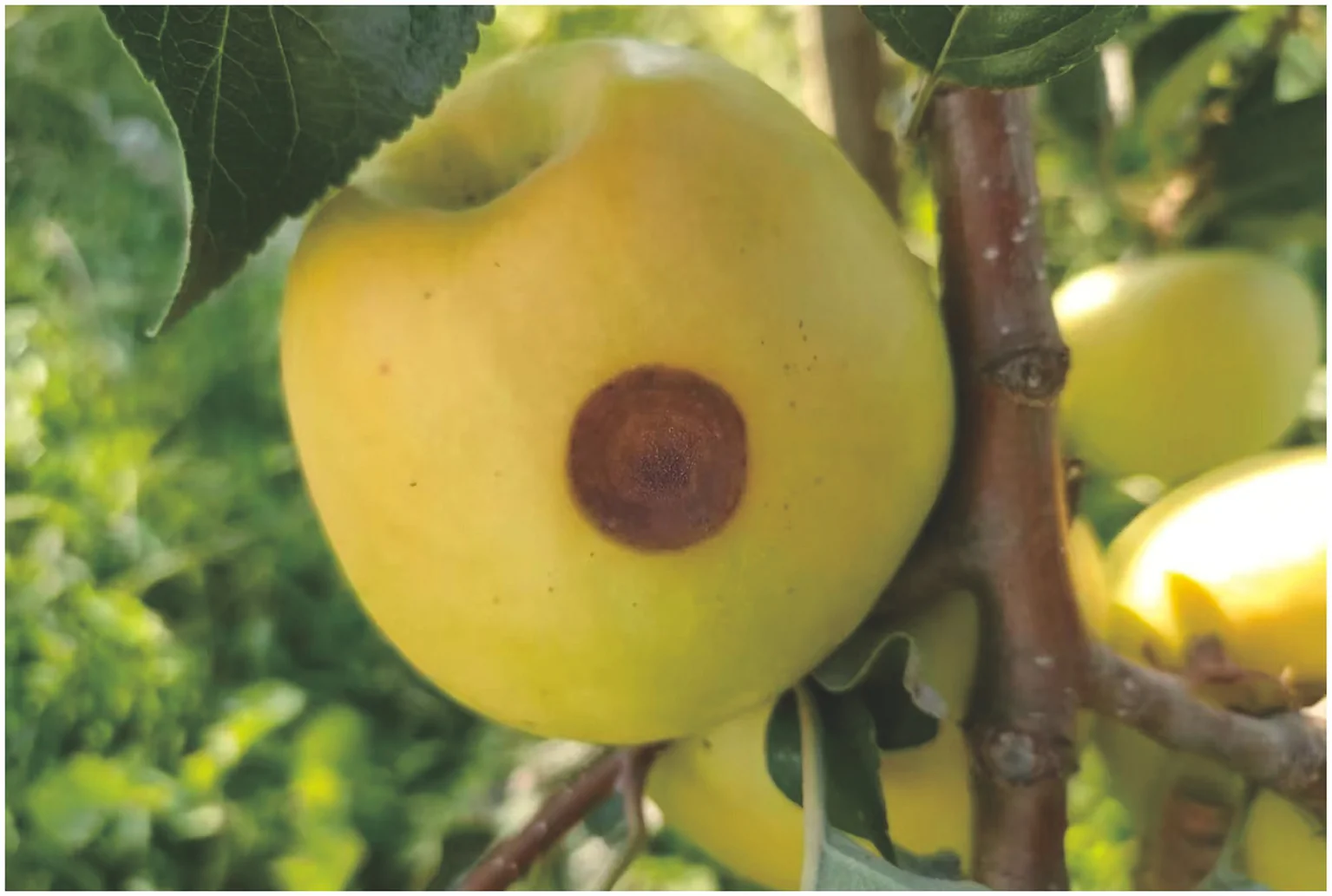
Symptoms of apple ring rot on fruits. Typical ring-shaped lesions on the fruit surface with gradual rot at the center of the lesions.

Fruit ring rot is one of the major contributors to apple yield loss and postharvest rot. The pathogen can usually infect fruits at the young fruit stage, but due to the latent infection characteristic, symptoms usually appear when the fruits are near maturity or during storage (Kohn and Hendrix, 1983; Marsberg et al., 2017). At the initial stage of infection, small brown spots appear around the fruit lenticels, and then the spots expand rapidly to form nearly circular lesions with a diameter of about 5–10 cm. The color of the lesions presents a typical alternating light and dark ring pattern. As the disease progresses, the pulp beneath the lesion gradually softens and decays, with the rot extending inward in a funnel-shaped pattern (Figure 2) (Guan et al., 2015; Zhang, 2013). In the late stage of the disease, a large number of small black dots, namely the conidiomata of *B. dothidea*, form on the surface of the lesions (Kim and Park, 2025). Under high humidity conditions, many conidia are released, becoming new inoculum sources (Liu et al., 2022). Current research has thoroughly described the macroscopic symptom sequence of fruit-associated ring rot, yet few studies explore the molecular regulatory mechanism governing the transition from latent infection to the onset of visible fruit lesions.

## 3 Pathogenesis of apple ring rot

**Figure 3:**
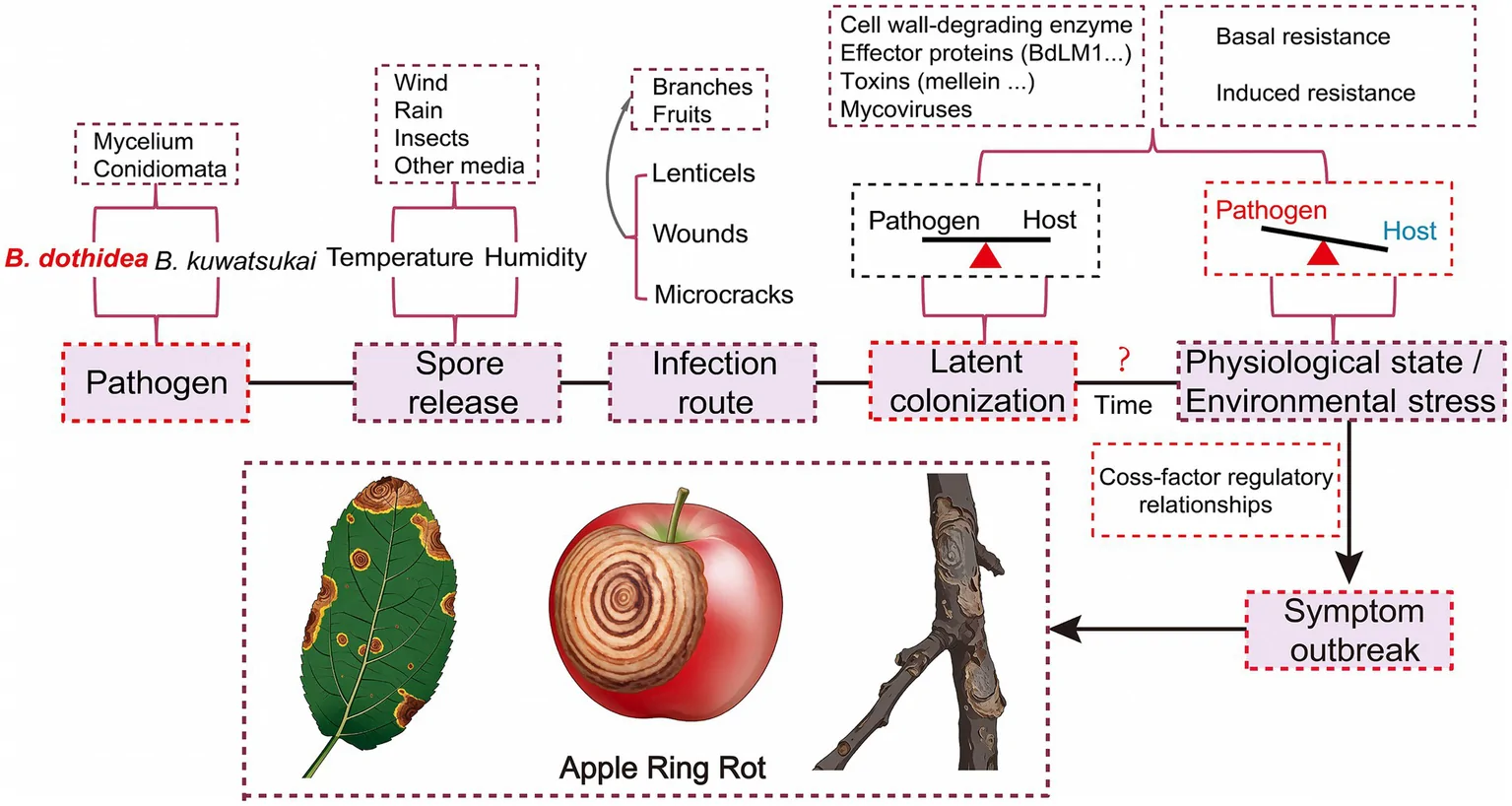
Pathogenesis of apple ring rot. The entire process follows the core pathway of “Pathogen → Spore release → Infection route → Latent colonization → Physiological state/Environmental stress → Symptom outbreak”. Pathogen: *B. dothidea* overwinters as mycelium and conidiomata in diseased branches and debris. Spore release: In spring, conidiomata produce and release conidia, dispersed by wind, rain, and insects as the primary inoculum. Infection route: Germinating spores invade through natural openings—lenticels on branches and lenticels plus surface microcracks on fruits. Latent colonization: The pathogen colonizes host tissues, maintaining host–pathogen equilibrium without visible symptoms. Symptom outbreak: Host vigor decline or environmental stress (e.g., drought) disrupts the equilibrium; the pathogen resumes active growth, rapidly colonizes and destroys tissue, producing ring rot symptoms on branches, fruits, and leaves. The symbol “?” highlights that synergistic and antagonistic cross-regulatory relationships among host, pathogen and environmental factors underlying latent infection remain uncharacterized.

The development of apple ring rot results from the interaction among pathogen infection, virulence factors, host responses, and environmental conditions. The infection cycle of this pathogen follows the core path: pathogen → spore release → infection route → latent colonization → physiological state/environmental stress → symptom outbreak (Figure 3). In this context, the pathogen is *B. dothidea*, while the infection cycle of *B. kuwatsukai* on *M. domestica* is presumed to follow a similar pattern, although it lacks systematic experimental verification.

### 3.1 Etiology of apple ring rot

#### 3.1.1 Pathogen

In European and American countries, a disease with similar symptoms is commonly referred to as apple white rot, and the pathogen is identified as *Botryosphaeria dothidea* (*B. dothidea*) has been identified as its causal agent (Jurick et al., 2013). Subsequent molecular and phylogenetic studies suggested that *B. berengeriana* f. sp. *Pyricola* is closely related to *B. dothidea* and supported its placement with the same species (Zhongdong et al., 2010). Furthermore, phylogenetic analyses showed that isolates associated with apple ring rot strains in China, Japan and South Korea and isolates associated with apple white rot in the United States were closely related, while pathogenicity assays demonstrated their ability to induce typical disease referred to as apple ring rot and apple white rot associated with closely related or conspecific *Botryosphaeria* taxa (Tang et al., 2012).

However, subsequent studies further found that apple ring rot is associated with at least two closely related species, *B. dothidea* and *Botryosphaeria kuwatsukai* (*B. kuwatsukai*), which have significant differences in morphology, pathogenicity and genome (Wang B. et al., 2021; Wang et al., 2018). *Botryosphaeria dothidea* has a relatively broad host range and has been reported from several woody and fruit crops, including *M. domestica*, pears and peaches, while *B. kuwatsukai* has a narrower host range and is primarily associated with *M. domestica* and pears. Comparative genomics analyses revealed that both species have undergone expansion of pathogenicity-related genes, but *B. kuwatsukai* has lost some genes related to secondary metabolism and cell wall-degrading enzymes due to host specialization, which may reflect host specialization (Wang et al., 2018). To date, how these genomic differences translate into differences in host range, latent infection capacity, lesion morphology, toxin production, fungicide sensitivity and field epidemic potential remains insufficiently tested. Most pathogenicity studies have focused on *B. dothidea*, while *B. kuwatsukai* is less characterized. Critically, the predominance of *B. dothidea* in current literature may skew our understanding of the disease’s epidemiology. While *B. kuwatsukai* is highly specialized, most pathogenicity studies focus on *B. dothidea*. This bias suggests that the specific virulence mechanisms of *B. kuwatsukai*—particularly its loss of secondary metabolism genes—remain underexplored.

#### 3.1.2 Pathogenic factors

The pathogenicity of fungal pathogens is mediated by multiple factors, including cell wall-degrading enzymes, effector proteins, and toxins, whereas mycoviruses can modulate fungal virulence (Belair et al., 2022). All virulence factors described below have been functionally characterized in *B. dothidea*; corresponding evidence for *B. kuwatsukai* is still lacking. Cell wall-degrading enzymes facilitate host tissue colonization by degrading components of the plant cell wall. *Botryosphaeria dothidea* produces enzymes involved in the degradation of cell wall components, including cellulases, pectinases, and hemicellulases. These enzymes can degrade the host cell wall, provide channels for hyphal expansion, and also provide nutrients for the pathogen (Restrepo-Leal et al., 2024). Several effector proteins have also been identified in *B. dothidea* and are associated with the suppression or modulation of host immune response (Zhang et al., 2021). For example, BdLM1, an effector protein containing a LysM domain, can bind chitin, protect *B. dothidea* hyphae from degradation by host chitinases, and also inhibit the host’s reactive oxygen species burst and immune response. Knockout of this gene significantly reduces the virulence of *B. dothidea* (Zhang et al., 2023). Notably, current functional validation of BdLM1 and other characterized effectors relies primarily on single-strain gene knockout assays, with most phenotypic evaluations performed using *in vitro* detached branches, fruits or leaves. Given that effector gene copy number and expression levels vary widely among wild strains with distinct genetic backgrounds, whether the virulence contributions of these effectors are universally conserved remains to be verified at the population level. These findings indicate that conserved effectors such as BdLM1 can serve as molecular diagnostic targets for the latent infection stage. By detecting the expression level of effector genes, early disease detection at the asymptomatic stage can be achieved, addressing the industrial pain point of difficult early diagnosis of latent infection.

The secretory proteins of *B. dothidea* include catalase (BdCatalase), cytochrome P450 family protein (BdCYP52A4) and subtilisin domain-containing proteins, etc. These proteins may help *B. dothidea* clear reactive oxygen species produced by the host and inhibit the host’s defense response, thereby promoting pathogen infection (Wang et al., 2024). This set of antioxidant secretory proteins is functionally unified in counteracting host oxidative burst, yet existing work only provides individual functional inference without comparative analysis of their relative contribution to ROS scavenging during latent versus active infection stages. Moreover, the cutinase gene *Bdo_10846* has been shown to contribute to the pathogenicity of *B. dothidea*. Dong B. Z. et al. (2021) reported that deletion of *Bdo_10846* gene reduced cutinase activity and fungal virulence, supporting its role in *M. domestica* infection and the development of wart-like symptoms. Even though individual secreted virulence factors have been characterized, few studies have dissected the synergistic effects among these effector proteins, and how they coordinate to complete latent-stage colonization remains poorly understood.

Toxins are important pathogenic factors of pathogens. A study identified mellein, an important toxin of *B. dothidea*, using UPLC-MS/MS technology. This toxin can cause host cell death, inhibit the host’s immune response, and play a key role in lesion expansion (Li et al., 2021). Further studies are needed to clarify its specific effects on host cell viability, immune responses, and lesion development. In addition, mycoviruses can also affect the virulence of *B. dothidea* (Wang et al., 2014). Hypovirulent strains carrying dsRNA viruses BdCV1 and BdVV2 have been associated with reduced pathogenicity, suggesting that mycovirus-mediated modulation of fungal physiology may influence disease development (Cong et al., 2025). These findings highlight the potential of mycoviruses as biological resources for the development of alternative strategies for controlling apple ring rot. Nevertheless, most current studies on hypovirulence-associated mycoviruses are conducted using laboratory-isolated strains. Limited data are available regarding viral carrying frequency and horizontal transmission efficiency within field *B. dothidea* populations, which greatly restricts the reproducibility of their biocontrol performance under practical conditions.

### 3.2 Occurrence regularity of apple ring rot

#### 3.2.1 Infection cycle

*Botryosphaeria dothidea* is characterized by a latent infection phase in apple, during which the pathogen can colonize host tissues without producing visible symptoms. The pathogen primarily survives the winters as mycelium and conidiomata in lesions on diseased branches, and can also survive in diseased debris such as fallen fruit and diseased leaves. In the following spring, when the temperature is suitable and the humidity is high, the overwintered *B. dothidea* will release many conidia from the conidiomata. These spores are transmitted to the host surface through wind, rain, insects and other media. After germination, the spores invade the host tissue through natural openings such as lenticels and wounds. For branches, *B. dothidea* mainly invades through lenticels, while for fruits, microcracks are also important infection routes in addition to lenticels (Li et al., 2006). Studies have found that the lenticel area and the number of microcracks on the surface of apple fruits are significantly positively correlated with the susceptibility of fruits to *B. dothidea*. Fruit susceptibility to infection is associated with the characteristics of lenticels and the presence of microcracks, which may facilitate *B. dothidea* penetration (Guan et al., 2015). However, this correlation has not been translated into a causal framework: whether lenticel density can serve as a reliable predictor of field susceptibility across diverse cultivars remains to be validated, and systematic evaluation of lenticel traits as a breeding marker for resistance has yet to be pursued. After invading the host, *B. dothidea* does not cause symptoms immediately, but enters the latent infection stage. In this stage, the pathogen exists in the host tissue with low level of growth, the host’s immune system is not strongly activated, and both are in a state of equilibrium. This latent stage can last for a long time. In fruits, the latent stage can last from the young fruit stage until the ripening stage, for up to several months. When the host’s physiological state changes or environmental conditions become favorable, this balance is disrupted, and the pathogen transitions from a latent state to an active growth state, multiplying rapidly, destroying host tissue, and thus causing symptoms (Kim et al., 2001; Marsberg et al., 2017). In fruits, as fruits ripen, its firmness decreases, sugar content increases, and its defense capacity declines, allowing the pathogen to grow rapidly and cause rot symptoms (Kohn and Hendrix, 1983). On branches, when the tree’s vigor is weak or it is under stress from drought frost, the pathogen will become active and causes lesions to spread. However, the molecular basis of this host–pathogen equilibrium remains poorly characterized. New conidiomata form in the affected lesions, and at the end of the growing season, these release new conidia for secondary infection, thus completing the infection cycle (Xue et al., 2021). In addition, the sexual state of the pathogen can produce ascospores as a source of inoculum, but under natural conditions, asexual conidia are the primary and secondary source of inoculum (Marsberg et al., 2017; Parker, 1993). All latent onset and symptom outbreak events are driven by dynamic shifts in host internal physiology, pathogen virulence expression and ambient environmental conditions. This sophisticated environment-mediated interplay of host metabolic signals and pathogen virulence represents a major unresolved frontier in current mechanistic research.

#### 3.2.2 Influencing factors

The occurrence and development of apple ring rot are influenced by the interaction of environmental conditions, host characteristics and pathogen-related factors (Coakley et al., 1999; Miller, 2003). Among environmental factors, temperature and moisture conditions play important roles in pathogen development, spore germination infection and disease progression (Sutton, 1991; Xue et al., 2021). High moisture conditions favor spore germination and infection of *B. dothidea*, while rainfall can contribute to the dissemination of inoculum and create favorable conditions for infection (Liu et al., 2022). In addition, the optimal growth temperature of *B. dothidea* is 28 °C, so the disease develops the fastest in the high-temperature and high-humidity season in summer (Liu et al., 2022). Changes in temperature and precipitation patterns may also influence the epidemiology of apple ring rot. However, further field-based studies are needed to determine how climate change may alter disease risk and seasonal development (Xue et al., 2021). Host characteristics also influence susceptibility to apple ring rot. Apple cultivars differ considerably in their susceptibility to *B. dothidea*, with differences in disease incidence and severity reported among cultivars (Miller, 2003; Shen et al., 2019; Yu X. et al., 2022). In addition, to genetic resistance, tree vigor, nutritional status, and fruit development stage may influence the occurrence of the disease (Zhang, 2025; Li, 2026; Csihon et al., 2024). However, a direct comparison of studies is confounded by differing experimental conditions (e.g., detached-fruit mycelial plug vs. field spore spray inoculation, variable inoculum dosage, fruit developmental stages, pathogen isolates). Furthermore, the relative contribution of pre-formed structural barriers (e.g., cuticle thickness) versus inducible biochemical defenses (e.g., phenolic compound accumulation) to overall resistance remains a subject of debate, as their relative importance appears to vary with the developmental stage of the fruit and the specific *M. domestica* cultivar (Guan et al., 2015; Wang et al., 2024). Pathogen-related characteristics also contribute to disease development. There are differences in pathogenicity among different *B. dothidea* strains, and highly virulent strains can cause more severe diseases (Cong et al., 2025). In addition, the development of fungicide resistance represents an important challenge for chemical disease management. For example, resistance to benzimidazole fungicides such as carbendazim has appeared in *B. dothidea* populations in numerous producing regions, which has led to a decrease in the control effect of fungicides and the aggravation of disease occurrence (Chen et al., 2021; Fan et al., 2016; Wang L. et al., 2021). Nevertheless, most studies address environmental, host, and pathogen effects on apple ring rot separately, with limited quantitative analysis of their interactive effects. This gap restricts precise epidemic prediction and targeted management. A key challenge is linking environmental drivers to molecular responses in both host and pathogen, rather than treating disease as a purely epidemiological phenomenon.

## 4 Resistance mechanisms of apple against ring rot

**Figure 4:**
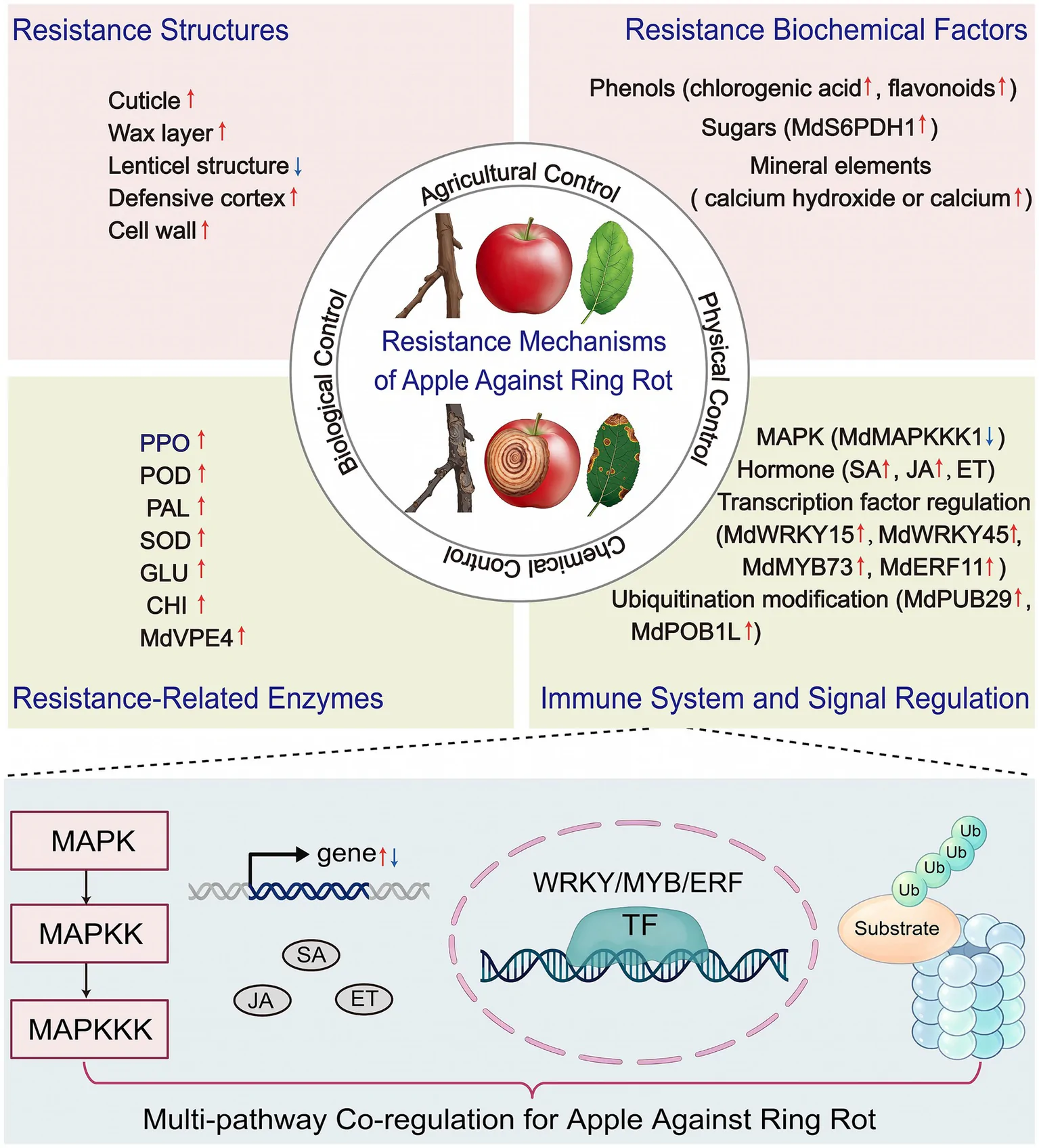
Resistance mechanisms of apple against ring rot. The resistance mechanisms of apple against apple ring rot are primarily divided into basal resistance and induced resistance. Basal resistance includes inherent physical structural barriers (e.g., cuticle, wax layer, wall) and endogenous antibacterial biochemical substances (e.g., phenols, sugars). Induced resistance consists of pathogen-activated defense enzymes (e.g., PPO, POD, PAL) and core immune signaling networks (e.g., MAPK cascade and hormone signaling pathways). These coordinated physical, metabolic and molecular defenses jointly limit latent fungal colonization and lesion expansion of *B. dothidea*. Arrows in the figure indicate the positive (↑) or negative (↓) regulatory effects of each component in the resistance response.

The resistance mechanism of apple against ring rot can be broadly classified into constitutive (basal) and induced defense responses. Basal resistance involves pre-existing structural and biochemical barriers that limit pathogen penetration and colonization. In contrast, induced resistance involves the activation of the defense response following pathogen recognition, including the activation of resistance-related enzymes and the regulation of immune signaling pathways (Figure 4) (Xu X. et al., 2022).

### 4.1 Basal resistance of apple ring rot

#### 4.1.1 Resistance structures

Resistance structures are the first physical barrier for hosts to resist pathogen infection, mainly including cuticle, wax layer, lenticel structure and cell wall (Hückelhoven, 2007; Winkler et al., 2022). The cuticle and wax layer form the outermost protective structures of the fruit epidermis and can restrict spore adhesion, germination, and pathogen penetration (Chen et al., 2022; Yeats and Rose, 2013). Studies have found that increased cuticular wax thickness positively correlates with enhanced fruit-level resistance against *B. dothidea* (Guan et al., 2015). Lenticels are important infection sites for *B. dothidea*, and their size and structural characteristics can influence fruit susceptibility. In different varieties during fruit development, the length of the lesion is positively correlated with the lenticels area (Guan et al., 2015). Yet this correlation does not establish causation: whether lenticel density can serve as a reliable screening marker for resistance breeding, independent of other co-varying fruit quality traits, has not been rigorously tested. In addition, the defensive cortex is a special structure formed by the host after infection. When *B. dothidea* invades the branch cortex, the healthy cells of the host divide rapidly to form a defensive cortex composed of 6–10 layers of tightly arranged cells. This cortex can physically isolate *B. dothidea* from healthy cells, prevent the expansion of the pathogen, and thus inhibit the development of the disease (Han et al., 2016). The cell wall is an important structural barrier to pathogen invasion. Cell-wall-related structural properties including thickness, lignin and cellulose contents are widely recognized to restrict hyphal invasion and pathogen spread across numerous apple-pathogen systems (Miedes et al., 2014). Cultivar-dependent variations in these wall-derived components are presumed to contribute to apple-fruit resistance toward *B. dothidea*. Numerous studies have verified the correlation between individual host structural traits and disease susceptibility, but researchers generally examine cuticle, lenticel and cell wall barriers separately and seldom quantify their synergistic protective capacity. The upstream genetic regulatory networks governing the development of these defensive tissue structures remain largely uncharacterized, representing a critical mechanistic gap in structural resistance research.

#### 4.1.2 Resistance biochemical factors

The biochemical factors in basal resistance include compounds present in host tissues before pathogen infection that can restrict pathogen growth or contribute to resistance. These compounds include phenolic metabolites, sugars, and mineral elements. These substances can inhibit the growth of pathogens and thus play a role in disease resistance. Phenolic compounds are important antibacterial substances in plants. The fruits of disease-resistant *M. domestica* varieties contain higher concentrations of phenolic compounds, such as chlorogenic acid, flavonoids, etc. These substances can inhibit the hyphal growth and spore germination of pathogens, thereby inhibiting pathogen infection (Nicholson and Hammerschmidt, 2003; Ribeiro et al., 2025). Studies have found that the contents of key metabolites in the phenylpropane metabolic pathway (such as chlorogenic acid) in disease-resistant varieties are significantly higher than those in susceptible varieties, and these substances can exert antibacterial effects by inhibiting the pathogen respiratory chain (He et al., 2022). In addition, the biocontrol strain *Pseudomonas syringae* B-1 (*P. syringae* B-1) can promote the accumulation of phenolic substances in *M. domestica* fruits, thereby significantly reducing the incidence of apple bitter pit (Sun et al., 2023). However, the role of phenolic compounds remains controversial. Several studies have demonstrated that phenolic substances themselves are not the main antibacterial agents, and the quinone substances formed by their oxidation are the key disease-resistant factors (Bangerth et al., 1972). This discrepancy may arise from differences in detection methods (e.g., total phenol quantification vs. specific quinone tracking) or the specific developmental stage of the fruit analyzed. Sorbitol has also been associated with differences in apple resistance to *B. dothidea*. Recent studies have found that the sorbitol content in disease-resistant *M. domestica* varieties is significantly higher than that in susceptible varieties. Sorbitol can activate the salicylic acid (SA) signaling pathway and improve the host’s immune ability. Overexpression of the sorbitol synthase gene *MdS6PDH1* can significantly improve the resistance of apple to ring rot, while silencing this gene leads to decreased resistance (He et al., 2022). Notably, sugars exhibit dual functionality: they act as carbon nutrients for invading pathogens and concurrently function as signaling mediators governing host immunity. This duality underscores an intricate metabolic trade-off whose underlying mechanisms remain largely elusive. Mineral elements also play important roles in resistance (Cao, 2018). In field trials, apple fruits treated with calcium hydroxide or calcium silicate showed reduced lesion size after inoculation with *B. dothidea* spores (Biggs, 2004). Current biochemical studies on apple basal resistance still have unresolved debates over the antifungal efficiencies of phenols and their oxidized quinone derivatives. Besides, most existing researches only analyse the defensive function of phenols, soluble sugars and mineral elements in isolation. Hence, follow-up research needs to systematically resolve the synergistic crosstalk among phenolic metabolism, sugar signal transduction and mineral ion homeostasis during *B. dothidea* infection, which can supplement the theoretical system of apple biochemical resistance.

### 4.2 Induced resistance of apple ring rot

#### 4.2.1 Resistance-related enzymes

When the host is infected by the pathogen, a large number of resistance-related enzymes are induced. These enzymes can improve the host’s disease resistance by scavenging reactive oxygen species (ROS), degrading the pathogen’s cell wall, synthesizing antibacterial substances. As an exogenous biocontrol elicitor, *P. syringae* B-1 is able to trigger induced resistance in apple fruits, and it alleviates oxidative damage, activates a suite of defense-related enzymes, promotes the accumulation of defensive compounds, and upregulates defense gene expression, ultimately enhancing fruit resistance to *B. dothidea* (Sun et al., 2023). Polyphenol oxidase (PPO) and peroxidase (POD) are important oxidases, which can catalyze the oxidation of phenolic substances to produce quinone substances. These quinone substances have stronger antibacterial activity and can inhibit the growth of pathogens (Mayer, 2006; Wichers et al., 2003). At the same time, POD is also involved in lignin synthesis, increasing the hardness of the cell wall by catalyzing the polymerization of lignin monomers, thereby preventing the expansion of the pathogen (Passardi et al., 2005). This dual function of POD—bridging biochemical oxidation and structural reinforcement—highlights the functional overlap between induced biochemical resistance and basal structural resistance, yet most studies treat these two resistance layers as independent modules. Furthermore, phenylalanine ammonia-lyase (PAL) is a key enzyme in the phenylpropane metabolic pathway, which can catalyze the conversion of phenylalanine to cinnamic acid, and then synthesize antibacterial substances such as phenols, lignin and SA (Zhang and Liu, 2015). Studies have found that butylated hydroxytoluene (BHT), *P. syringae* B-1, etc., can significantly increase PAL activity, thereby improving the host’s disease resistance (Huang et al., 2021; Sun et al., 2023). ROS scavenging acts as the shared response of all these enzymes, which then drive distinct phenolic and lignin defense pathways. β-1,3-glucanase (GLU) and chitinase (CHI) are important pathogen cell wall-degrading enzymes, which can degrade β-1,3-glucan and chitin in the pathogen cell wall respectively, thereby destroying the pathogen cell wall and inhibiting pathogen growth. These two enzymes are also important components of pathogenesis-related proteins (PR proteins) and play important roles in the host’s disease resistance response. Studies have found that after pathogen infection, the activities of GLU and CHI in apples increase significantly, and exogenous inducers can further increase the activities of these two enzymes, thereby enhancing the host’s disease resistance (Zhang et al., 2016). However, given that *B. dothidea* effectors such as BdLM1 can mask chitin from host recognition the actual *in vivo* efficacy of chitinase against this pathogen during latent infection warrants re-evaluation. In addition, vacuolar processing enzyme 4 (MdVPE4) also plays an important role in resistance. MdVPE4 can regulate the expression of PR proteins. Overexpression of *MdVPE4* can significantly improve the host’s resistance, while silencing this gene leads to decreased resistance (Dong et al., 2022). Although current physiological experiments have verified the induction patterns of these defense enzymes under single inducer or single pathogen treatment, most studies adopt isolated detection of individual enzyme activities and lack systematic comparative analysis of synergistic or antagonistic relationships among PPO, POD, PAL, GLU and CHI. Moreover, the threshold concentration of exogenous elicitors required to sustain long-term enzyme activation remains controversial across different apple genotypes, and few reports have clarified how latent infection suppresses the continuous accumulation of these resistance enzymes in host tissues.

#### 4.2.2 Immune system and signal regulation

The apple immune system can recognize the molecular patterns of pathogens, and then activate downstream signaling pathways to regulate defense responses. Among them, the MAPK cascade, hormone signaling pathways and transcription factor regulation are the core parts. The MAPK cascade is the core pathway of plant innate immunity. For example, studies have found that apple MdMAPKKK1 plays an important role in resistance to *B. dothidea*. The pathogen can induce the expression of *MdMAPKKK1* and activate the MAPK cascade reaction; overexpression of *MdMAPKKK1* triggers pathogen-independent cell death, while silencing significantly reduces the disease resistance of apple callus and fruits. MdMAPKKK1 can interact with MdBSK1 and be phosphorylated by it. This phosphorylation process is crucial for maintaining disease resistance, and the two synergistically mediate the defense response of apples to ring rot (Wang et al., 2022). Elucidation of such immune signaling modules lays a theoretical foundation for molecular breeding and the development of targeted disease management strategies against apple ring rot. The plant hormones such as SA, jasmonic acid (JA) and ethylene (ET) also play important roles in plant immune responses (Diaz-Vivancos et al., 2017; Peng et al., 2021; Zhang P. et al., 2025). Overexpression of the hexokinase-encoding gene MdHXK1 improves apple resistance to *B. dothidea* in calli, leaves and fruits. The underlying mechanism involves upregulating the expression of genes related to SA synthesis and signaling, as well as increasing the superoxide (O₂^−^) production rate and the hydrogen peroxide (H₂O₂) content (Yu J. Q. et al., 2022). Studies have found that MdSYP121 is a negative regulator of apple resistance to ring rot. Down-regulating its expression can significantly enhance the resistance of apples to *B. dothidea* by activating the SA signaling pathway and enhancing redox defense responses (He et al., 2018). Accordingly, these negative regulators constitute promising targets for CRISPR gene-editing breeding, as their knockout can elevate disease resistance with little compromise to fruit quality. Pathogen infection triggers upregulation of *MdABCI17*, which stimulates JA biosynthetic genes to boost JA accumulation and activate JA-responsive defensive cascades that improve apple immunity (Xiang et al., 2024). Existing research separately delineates *MdSYP121*-dependent SA and *MdABCI17*-dependent JA immune modules, yet the hormonal crosstalk between these two pathways during *B. dothidea* infection remains unelucidated. ET exerts dual regulatory effects on apple resistance to *B. dothidea*. The ET-responsive gene *MdERF11* and hexokinase-encoding gene *MdHXK1* positively drive antifungal immunity (Wang et al., 2020; Yu J. Q. et al., 2022). Nevertheless, infection-triggered excess ET aggravates fruit disease development (Qiu, 2017; He et al., 2018). This contradiction suggests that the timing and concentration of ET signaling are critical—acting as a defense signal in the early stages but potentially aiding the necrotrophic pathogen in later stages. Most current studies rely on transient expression assays which may not capture this temporal dynamic, leading to conflicting conclusions about whether ET is a positive or negative regulator. Accordingly, the exact regulatory modes of ET-mediated defense remain to be elucidated. In addition, multiple transcription factors such as WRKY, MYB and ERF modulate disease resistance by regulating the hormone signaling pathways. For example, MdWRKY15 can bind to the promoter of *MdICS1* to activate its transcription, thereby increasing SA accumulation and the expression of related genes, and further improving the resistance of apples to *B. dothidea* (Zhao et al., 2020). MdWRKY45 is a transcription factor localized in the nucleus with transcriptional activity and induced by *B. dothidea*. It positively regulates the resistance of Fuji apples to ring rot by regulating the contents of SA and jasmonic acid (JA) and ROS burst, and activating defense responses by binding to the promoters of genes such as *MdPR1* and *MdJAZ1* (Wang et al., 2024). MdMYB73 can improve the host’s resistance by activating the SA pathway, and can interact with MdWRKY31 to jointly regulate the defense response (Gu et al., 2021). In addition, MdERF11 is infection-inducible ERF transcription factor that positively boost apple resistance to *B. dothidea* (Wang et al., 2020). Deciphering these transcription factor-mediated regulatory networks provides abundant candidate genes for resistance breeding and inspires the exploitation of hormone-based elicitors for disease control; future research should further uncover the interwoven regulatory crosstalk among different transcription factors to facilitate the translational application of molecular findings in orchard management.

Beyond TF-mediated hormonal control, ubiquitination modification also plays an important role in resistance regulation. For example, the U-box E3 ligase MdPUB29 is a positive regulator of resistance to *B. dothidea*, which can activate the SA pathway and positively regulate the immune defense response of apples to ring rot (Han et al., 2019). The BTB-BACK domain E3 ubiquitin ligase MdPOB1L is also a positive regulator of the defense response against *B. dothidea*. It is induced by pathogen infection and SA, and can interact with MdNPR1 in the nucleus, promote the ubiquitination and degradation of MdNPR1 through the ubiquitin-proteasome pathway, and then activate the SA signaling pathway and up-regulate the expression of pathogenesis-related genes, ultimately enhancing the resistance of apples to ring rot (Zhang F. et al., 2025). Unraveling these ubiquitination-mediated immune mechanisms expands the pool of candidate genes for disease-resistance breeding and offers new insights into immune signal fine-tuning. Future research should focus on dissecting the extensive ubiquitination interactome and its crosstalk with other immune pathways to accelerate the translation of post-translational modification research into practical apple ring rot management strategies.

To sum up, existing research has separately identified multiple immune regulatory modules including MAPK cascade, hormone signaling pathways, transcription factor regulation and ubiquitin modification. Characterization of these immune regulatory pathways provides molecular underpinnings for four categories of disease-control technologies. However, most studies only focus on single-gene functional verification under transient infection conditions. There is a lack of systematic cross-comparison of crosstalk among these core signal networks, and it remains unclear whether the contradictory regulatory effects of partial hormones and transcription factors reported in different studies stem from cultivar differences or distinct pathogen inoculation dosages, which creates major knowledge gaps for constructing a complete apple induced immune regulatory network against ring rot.

## 5 Control measures of apple ring rot

Currently, a comprehensive integrated management framework consisting of agronomic, physical, chemical and biological tactics has been established to mitigate apple ring rot. In recent years, the advancement of synergistic green control strategies that integrate all four categories of management measures has delivered innovative technical approaches for the sustainable suppression of this fungal disease. The main advantages, field application difficulties and inherent limitations of all disease management approaches are summarized in Table 1.

**Table 1:** Main advantages, field application difficulties and limitations of four major control strategies against apple ring rot.

Control category	Representative measures	Main advantages	Field application difficulties & inherent limitations
Agricultural control	Rational fertilization and pruning, sanitation clearing of diseased tissues, heterologous cross-pollination.	Low cost, zero chemical residue, long-term reduction of primary pathogen inoculum.	Only preventive effect, relies on standardized long-term orchard management, hard to implement for scattered smallholder orchards.
Physical control	Double-layer fruit bagging, confocal Raman & NIR early detection, LEO@ZIF-8 postharvest chitosan fresh-keeping film.	Fully green, no fruit or environmental pollution, bagging improves fruit commodity quality.	Bagging only protects fruits, cannot control branch disease, detection equipment costly and not portable for field use, composite film only suitable for postharvest storage.
Chemical control	Traditional fungicides, novel systemic fungicides, nano-pesticide mixtures, plant resistance elicitors, botanical antifungal compounds.	Fast acting, broad-spectrum, effective for emergency epidemic intervention.	Long-term single use accelerates pathogen fungicide resistance, most botanical agents show drastically reduced efficacy under field conditions with high extraction costs, partial fungicides carry phytotoxicity risks.
Biological control	Antagonistic bacteria (*P. syringae, Bacillus* spp.), molecular marker-assisted breeding, CRISPR gene editing, HIGS, cross-kingdom RNAi, hypovirulent mycoviruses.	Safe and eco-friendly, genetic modification achieves durable host resistance, HIGS targets pathogen genes with high specificity.	Biocontrol strains have unstable colonization under fluctuating temperature/humidity, gene-edited apple materials face strict cultivation regulations, HIGS/mycovirus technologies remain confined to laboratory research.

### 5.1 Agricultural control

Agricultural control is the basis of integrated control. Orchard management is also an important part of agricultural control. Reasonable fertilization, especially calcium application, combined with appropriate pruning, maintaining ventilation and light transmission in the orchard, and timely removal of diseased residues such as diseased branches and fruits can reduce the primary inoculum source of pathogens and lower the incidence of fungal diseases such as ring rot (Zhang, 2025; Li, 2026; Csihon et al., 2024). In addition, cross-pollination with heterologous pollen can effectively improve the disease resistance of apple fruits, thereby improving fruit quality and enhancing their resistance to diseases such as ring rot (Chen et al., 2025). Nevertheless, agricultural management measures only exert preventive effects rather than therapeutic effects on existing infections. Moreover, the implementation of standardized orchard maintenance relies on long-term, normative field management systems, which is difficult to popularize and implement in scattered small-scale individual orchards across major apple-producing regions.

### 5.2 Physical control

Physical control is an environmentally friendly method of control. Studies have found that wrapping fruits with double-layer paper bags can effectively reduce the lenticels area and increase the thickness of the cuticular wax, thereby improving the disease resistance of fruits (Guan et al., 2015). However, this physical barrier strategy only blocks surface pathogen penetration on fruits and cannot mitigate branch infections or eliminate latent pathogens that have already colonized fruit tissues, functioning solely as a prophylactic rather than therapeutic measure. To clarify the interaction mechanism between *B. dothidea* infection process and apple cell defense, a study proposed a label-free and high-throughput imaging method based on confocal Raman microspectroscopy imaging technology combined with the multivariate curve resolution-alternating least squares algorithm (MCR-ALS) to analyze the temporal and spatial changes of peel cell wall components. At the same time, a near-infrared nondestructive detection method was developed, which can achieve early and accurate identification of ring rot-infected fruits with a classification accuracy of 100% (Li M. et al., 2024). While these diagnostic technologies deliver excellent accuracy under controlled laboratory conditions, their dependence on benchtop equipment restricts large-scale on-site deployment in commercial orchards. Furthermore, most validation is performed on artificially inoculated samples, and detection reliability for naturally infected asymptomatic fruit under complex field conditions remains underexplored. Recent studies have found that LEO@ZIF-8/chitosan composite film can delay the development of postharvest apple ring rot and reduce associated damage, indicating their potential as an active packaging strategy for postharvest disease management (Tong et al., 2026). Nevertheless, as a postharvest storage technology, this approach only suppresses disease development during the storage period and cannot reduce preharvest infection pressure in orchards; it must be integrated with preharvest field control measures to achieve full-cycle disease management. Collectively, physical control technologies target specific stages of disease development but lack cross-stage synergistic effects. Their practical application is constrained by scenario specificity and equipment cost, positioning them as complementary rather than standalone solutions for comprehensive apple ring rot management.

### 5.3 Chemical control

Chemical control is currently the main means of apple ring rot control. Traditional fungicides, such as carbendazim, mancozeb and thiophanate-methyl, have played important roles in the control of ring rot. These fungicides can be sprayed during the pathogen infection period to kill spores and inhibit spore germination, thereby reducing infection (Liu et al., 2010; Na et al., 2009; Qiong and Wen-Zhe, 2012; Zhong-Gang et al., 2005). Additional research has shown that the combined application of silver nanoparticles synthesized from holly leaves and mancozeb can synergistically inhibit the growth of *B. dothidea* and alleviate the problems of pesticide residues and pathogen resistance (Xu M. et al., 2022). Although conventional fungicides provide rapid and reliable control efficacy, their decades-long extensive use has driven widespread fungicide resistance evolution in field *B. dothidea* populations across multiple apple-growing regions (Fan et al., 2022; Wang L. et al., 2021). The nano-fungicide combination strategy partially mitigates this problem, but its long-term field stability and ecological safety still lack systematic multi-year evaluation. New fungicides, such as pyraclostrobin, tebuconazole and flusilazole, have higher control effects and stronger systemic absorption, which can better control the disease. Studies have found that pyraclostrobin has a good inhibitory effect on *B. dothidea* with an EC₅₀ value of 3.0870 μg/mL. Field trials have shown that pyraclostrobin at 200 and 250 g a.i./ha can achieve a control effect of more than 80%, and has a longer duration compared with traditional fungicides (Fan et al., 2019). Triazole fungicides such as tebuconazole can also effectively control ring rot and can simultaneously control other fungal diseases (Fan et al., 2016). Compared with traditional agents, these systemic fungicides have higher specific activity, but most efficacy data come from single-site or single-year field trials. Their cross-regional reproducibility under variable climatic conditions and pathogen population structures remains insufficiently validated, and their long-term contribution to resistance development requires continuous monitoring.

Induced resistance is also an important means of chemical control. By using inducers to induce the host’s own defense response, the host’s disease resistance can be improved. For example, the plant activator BHT can induce the resistance of apple fruits, improve the activity of defense enzymes, increase the content of SA, activate the SA signaling pathway, and thus significantly inhibit the infection of *B. dothidea* (Huang et al., 2021). This host immunity-oriented strategy imposes lower resistance selection pressure on pathogens than direct-killing fungicides, but its efficacy is highly dependent on application timing and host physiological status, resulting in less stable field performance compared with conventional fungicides. Natural compounds are also a research hotspot in recent years. For example, berberine, an alkaloid extracted from Coptis chinensis, has a good inhibitory effect on *B. dothidea*. Studies have found that berberine can inhibit the hyphal growth of the pathogen, destroy the pathogen’s cell membrane, and inhibit the pathogen’s energy metabolism, with an EC₅₀ value of 8.4–9.8 μg/mL and a field control effect of 78.27%. Moreover, this natural fungicide is safe and non-toxic and will not cause pesticide residues (Li P. et al., 2024). Studies have found that dimethyl trisulfide (DT), the main component of Chinese chive volatiles, can inhibit the growth of pathogens and induce the expression of host defense genes, with a significant control effect on ring rot (Sun et al., 2022). Through transcriptomic, metabolomic and physiological analyses, it was found that diaporthein B (DTB), a sesquiterpenoid compound isolated from a marine-derived fungus, can interfere with mitochondrial function and disrupt the tricarboxylic acid cycle and oxidative phosphorylation process. This interference leads to an increase in reactive oxygen species content, which in turn triggers lipid peroxidation, and ultimately inhibits the hyphal growth of *B. dothidea* (Du et al., 2024). Another study found that (Z)-ligustilide extracted from *Saposhnikovia divaricata* has a strong ability to inhibit the growth of *B. dothidea* and has great potential in preventing apple ring rot (Tong et al., 2025). These plant - and microbe-derived compounds have prominent low-toxicity advantages, but almost all efficacy data are derived from laboratory assays and small-scale field tests. High raw material extraction costs, fluctuating field efficacy under variable environmental conditions, and potential phytotoxicity risks collectively limit their large-scale commercial application. In addition, inconsistent bioassay protocols across independent studies make direct cross-comparison of their antifungal potency unreliable. Overall, chemical control remains the cornerstone of apple ring rot management, but an inherent trade-off exists among control efficacy, resistance risk and environmental safety. Shifting from traditional single-agent application to diversified integrated chemical strategies combining systemic fungicides, resistance inducers and natural compounds is a clear development trend, yet a standardized field-relevant efficacy evaluation system is still lacking to guide practical production.

### 5.4 Biological control

Biological control has been a research hotspot for many years, with the advantages of environmental friendliness and safety, mainly including biocontrol bacteria, disease-resistant breeding and new molecular biology technologies (Droby et al., 2009; Huang et al., 2022; Sharma et al., 2009). The application of antagonistic microbes relies on a dual strategy of direct antifungal activity and indirect induced resistance. For instance, the biocontrol bacterium *P. syringae* B-1 isolated from the apple surfaces can significantly inhibit the hyphal growth and spore germination of *B. dothidea*. In apple fruit, treatment with strain B-1 reduced the incidence of apple ring rot and lesion diameter by 41.2 and 90.2%, respectively. Its biocontrol activity was associated with reduced oxidative damage, increased antioxidant and defense-related enzyme activities, enhanced accumulation of phenolics, lignin, and SA, and upregulation of defense-related genes, including PR1, PR5, GLU, and CHI, as well as genes involved in SA biosynthesis (SID2 and PAD4) (Sun et al., 2023). In addition, Bacillus species such as *Bacillus amyloliquefaciens*, *Bacillus subtilis* and *Bacillus halotolerans* also have good inhibitory effects on *B. dothidea*. They can produce antibacterial substances to inhibit the growth of pathogens, and can also colonize the fruit surface and occupy ecological niches to prevent pathogen infection (Calvo et al., 2017; Fan et al., 2017; Yuan et al., 2022). However, the efficacy of these microbial agents is highly context-dependent, revealing a significant gap between controlled experiments and field application. While these strains demonstrate potent antagonistic activity *in vitro*, their performance in commercial orchards is often inconsistent. The colonization efficiency of these beneficial microbes is notoriously unstable under fluctuating field conditions, particularly variations in temperature and humidity, which severely limits their reliability as a standalone control measure.

In recent years, advancements in molecular biology have opened new avenues for genetic resistance, moving from marker-assisted selection to precise gene editing. The researchers have located QTL loci for apple ring rot resistance through multi-omics methods and developed 10 effective molecular markers, providing tools for molecular marker-assisted breeding (Shen et al., 2019). More recently, gene editing technology has also been applied to disease-resistant breeding. For example, editing the negative resistance regulator gene *MdCNGC2* through CRISPR/Cas9 can significantly improve the resistance of apples to ring rot (Zhou et al., 2020). Studies also highlight the role of apple vacuolar processing enzyme 4 (MdVPE4), where silencing reduces resistance while overexpression enhances it (Dong et al., 2022). Furthermore, the E2-like enzyme Atg3 plays an important role in various growth and development processes and pathogenic mechanisms of *B. dothidea*, so it is expected to become a potential target for the development of new fungicides (Han et al., 2025). Host-Induced Gene Silencing (HIGS) harnesses the host’s endogenous RNAi machinery against the pathogen. Studies have found that wild apple miR159a can be transported into *B. dothidea* through extracellular vesicles, silencing the pathogen’s sugar transporter gene *BdSTP*, thereby inhibiting the growth of the pathogen. This is a natural cross-kingdom RNAi mechanism. Based on this mechanism, small artificial RNAs (sRNAs) have been developed, which can target the pathogenic genes of the pathogen and significantly improve the host’s resistance, providing a new strategy for disease control (Yu et al., 2024). Despite the immense theoretical potential of these genetic and molecular strategies, their practical implementation is currently bottlenecked by regulatory and technical barriers. While gene-edited materials like *MdCNGC2* mutants show promise, their commercial application is heavily restricted by strict agricultural biosafety supervision policies globally. Similarly, advanced technologies like HIGS and hypovirulent mycoviruses remain confined to laboratory research and cannot be applied in actual field production at present. Consequently, biological control currently faces a dichotomy: traditional biocontrol agents struggle with environmental stability, while next-generation genetic solutions struggle with regulatory acceptance and delivery mechanisms.

## 6 Problems and prospects

After years of research, significant progress has been made in the study of apple ring rot, and we have an in-depth understanding of the pathogen classification, pathogenesis, host resistance mechanism and control technologies. However, multiple core bottlenecks and unresolved research gaps persist across this field, restricting the sustainable prevention of this destructive disease.

First, the latent infection mechanism of the pathogen is still unclear. The latent infection of *B. dothidea* is an important reason why ring rot is difficult to control. The pathogen can remain latent in host tissues without causing symptoms, but we still know little about how the pathogen evades host immune recognition, how it maintains the latent state, and how it transforms from the latent state to the pathogenic state. Future research needs to use omics technologies such as transcriptomics and metabolomics to deeply analyze the intermolecular regulatory networks among host, pathogen and environmental cues during the latent stage, so as to provide a theoretical basis for early disease diagnosis. Second, abundant contradictions and unsolved puzzles still exist regarding the regulatory crosstalk between multi-layered apple defense pathways. Current studies mostly separately characterize structural barriers, antibacterial metabolites, defense enzymes and immune signaling cascades, while few systematic investigations quantitatively dissect their synergistic or antagonistic interactions upon pathogen infection. These unresolved crosstalk mechanisms across physical, biochemical and molecular defense layers constitute a major mechanistic gap, and future multi-omics integrated research is urgently required to decode these complex interactive regulatory networks. Third, the breeding of disease-resistant varieties is still progressing slowly. Although we have found some disease-resistant germplasm resources, there are still few studies on the cloning and function of resistance genes. At present, no major-effect ring rot resistance gene has been cloned through map-based cloning approaches, which leads to the slow progress of molecular marker-assisted breeding and gene editing breeding. Future research needs to strengthen the mining of resistance genes, use technologies such as genome-wide association analysis and map-based cloning to clone key resistance genes, so as to provide genetic resources for disease-resistant breeding. Fourth, the problem of pathogen drug resistance is becoming increasingly serious. Long-term use of chemical fungicides has led to the development of drug resistance in *B. dothidea* populations, and many traditional fungicides have lost control effects. Future research needs to strengthen the study of pathogen drug resistance mechanisms, establish a drug resistance monitoring system, and develop new fungicides, especially natural fungicides, as well as reasonable medication strategies to delay the emergence of drug resistance. Finally, the application of biological control technologies is still limited. Although we have screened many biocontrol microorganisms, most available efficacy data are derived from laboratory *in vitro* assays rather than field or commercial orchard trials. This lack of field validation hinders the assessment of practical reliability and the promotion of these biocontrol agents in production. Future research should prioritize field-scale validation of biocontrol agents under commercial orchard conditions to assess their practical reliability. Furthermore, the impact of climate change on apple ring rot also needs further study. With global warming, the increase in temperature will prolong the growth season of the pathogen and also lead to weak tree vigor, which may aggravate the occurrence of ring rot. Future research needs to evaluate the impact of climate change on the occurrence of ring rot and develop adaptive control strategies to meet the challenges brought by climate change.

Collectively, apple ring rot constitutes a persistent threat to the global apple industry. Although significant progress has been made, we still face many challenges. Future research should prioritize the elucidation of environment-mediated pathogen–host interaction mechanisms, disease-resistant breeding, and green control technologies to achieve sustainable control and ensure the healthy development of the industry.
